# Permeation of Dopamine Sulfate through the Blood-Brain Barrier

**DOI:** 10.1371/journal.pone.0133904

**Published:** 2015-07-24

**Authors:** Tina Suominen, T. Petteri Piepponen, Risto Kostiainen

**Affiliations:** 1 Division of Pharmaceutical Chemistry and Technology, Faculty of Pharmacy, University of Helsinki, Helsinki, Finland; 2 Division of Pharmacology and Pharmacotherapy, Faculty of Pharmacy, University of Helsinki, Helsinki, Finland; Institute of Neurology (Edinger-Institute), GERMANY

## Abstract

Dopamine sulfate (DA-3- and DA-4-S) have been determined in the human brain, but it is unclear whether they are locally formed in the central nervous system (CNS), or transported into the CNS from peripheral sources. In the current study, permeation of the blood-brain barrier (BBB) by DA-S was studied by injecting ^13^C_6_-labelled regioisomers of DA-S (^13^DA-3-S and ^13^DA-4-S) and dopamine (DA) subcutaneously (s.c.) in anesthetized rats, then analyzing brain microdialysis and plasma samples by UPLC-MS/MS. The results in the microdialysis samples demonstrated that brain concentrations of ^13^DA-S regioisomers clearly increased after the s.c. injections. The concentration of DA did not change, indicating the permeation of DA-S through an intact BBB. The analysis of plasma samples, however, showed that DA-S only permeates the BBB to a small extent, as the concentrations in plasma were substantially higher than in the microdialysis samples. The results also showed that the concentrations of DA-3-S were around three times higher than the concentrations of DA-4-S in rat brain, as well as in the plasma samples after the s.c. injections, indicating that DA-3-S and DA-4-S permeate the BBB with similar efficiency. The fate of ^13^DA-S in brain was followed by monitoring ^13^C_6_-labelled DA-S hydrolysis products, i.e. ^13^DA and its common metabolites; however, no ^13^C_6_-labelled products were detected. This suggests that DA-S either permeates through the BBB back to the peripheral circulation or is dissociated or metabolized by unexpected mechanisms.

## Introduction

The monoamine neurotransmitters dopamine (DA) and serotonin (5-HT) are involved in several physiological processes in the brain [1–3]. Both DA and 5-HT are metabolized by monoamine oxidase (MAO) to the phase I metabolites 3,4-dihydroxyphenylacetic acid (DOPAC) and 5-hydroxyindoleacetic acid (5-HIAA), respectively. DOPAC is further metabolized to homovanillic acid (HVA) by catechol-O-methyltransferase (COMT). Both DA and 5-HT, and their respective metabolites, can undergo conjugation with glucuronic acid or sulfonate mediated by catalysis with UDP-glucuronosyltransferases (UGTs) and sulfotransferases (SULTs), respectively. The glucuronide and sulfate conjugates have been considered inactive, but recently the activity of glucuronide and sulfate conjugates of a few other substances has been shown. For example, the glucuronide or sulfate conjugates of dehydroepiandrosterone, pregnenolone [4,5], and morphine [6] are thought to be more active than the parent compounds in modulating their pharmacological effects in the central nervous system (CNS).

The pharmacological properties of the sulfonate conjugates of neurotransmitters are not well known. Some pharmacological properties of DA-S have been reported: intraventricularly injected DA-S has been shown to trigger severe convulsions in conscious rats, which did not occur when unconjugated dopamine was administered [7,8]. DA-S has been shown to bind to the GABA receptor, but not to the dopamine receptors (D1 or D2) [7,9]. The regioisomer DA-3-S has also been shown to have an inhibitory effect on aldosterone secretion in bovine cells in vitro [10].

The BBB is a physical and biochemical barrier between the blood and the brain, which prevents entry into the brain of most drugs and endogenous compounds from the blood. Only small lipophilic compounds can diffuse passively through the BBB, while other compounds are usually able to cross the BBB only with the help of carrier proteins [11,12]. The BBB is formed by the brain capillary endothelial cells, and in addition to this physical barrier there are several transporter proteins located in the BBB, e.g. ATP-binding cassette (ABC) transporters such as P-glycoprotein (P-gp), multidrug resistance proteins (MRP in humans; Mrp in rodents), breast cancer resistance proteins (BCRP), and solute carriers (SLC:s), such as the organic anion-transporting polypeptides (OATPs in humans, Oatps in rodents), that are involved in the regulation of substances in the central nervous system and the brain [11,13–15]. Some sulfate conjugates are known substrates of different OATP/Oatp-transporters, such as the steroid conjugates estrone-3-sulfate, estradiol-17β-glucuronide, and dehydroepiandrosterone sulfate (DHEA-S) [14,16]. DHEA-S has been shown to permeate the BBB with the help of these transporter-proteins, although the transport is mainly from the brain to the blood [16,17]. The sulfonate conjugate of pregnenolone has also been shown to permeate the BBB [18].

Microdialysis is a reliable method to examine the physiological state of the brain, or to estimate drug transport to the brain across the BBB. A probe is inserted into a specific brain area, and sampling is accomplished by pumping a solution, isotonically matched to the medium being sampled, slowly through the probe and collecting it for analysis. The dialysis membrane is permeable to small molecules but not to macromolecules such as proteins. The small molecules are sampled by the probe, because the levels of neurotransmitters and metabolites are higher in the extracellular space than in the perfusion fluid [19,20]. The levels of most neurotransmitters in the brain are low and sensitive analysis techniques, such as mass spectrometry, must be utilized.

Only recently, sensitive mass spectrometric methods have been developed for the detection of intact phase II metabolites of monoamine neurotransmitters and neurosteroids in rodent and human brains [21–25]. Intact glucuronide conjugates of DA and 5-HT, and sulfonate conjugates of their phase I metabolites DOPAC, HVA, and 5-HIAA have been found in rat brain [24], and intact sulfates of 5-HIAA and DA in human brain [25]. Earlier analytical methods for the identification of sulfate and glucuronide conjugates of neurotransmitters involved a hydrolysis step [26–28], and did not provide identification of the conjugates with absolute certainty or information about the site of conjugation. Whether the determined sulfate and glucuronide conjugates are locally formed in the brain or originate in peripheral sources is still unknown. Low mRNA levels of the UGTs that are thought to be involved in the conjugation of DA and 5-HT (e.g. UGTs 1A6 and 2B7) have been reported in rat and human brain, which makes local formation an alternative [29–31]. In addition, SULTs responsible for the sulfonation of catecholamines have been found in human and rat brain [32]. However, the formation of DA and 5-HT conjugates in peripheral organs and their transport to the brain through the blood-brain barrier (BBB) cannot be ruled out.

**Fig 1:**
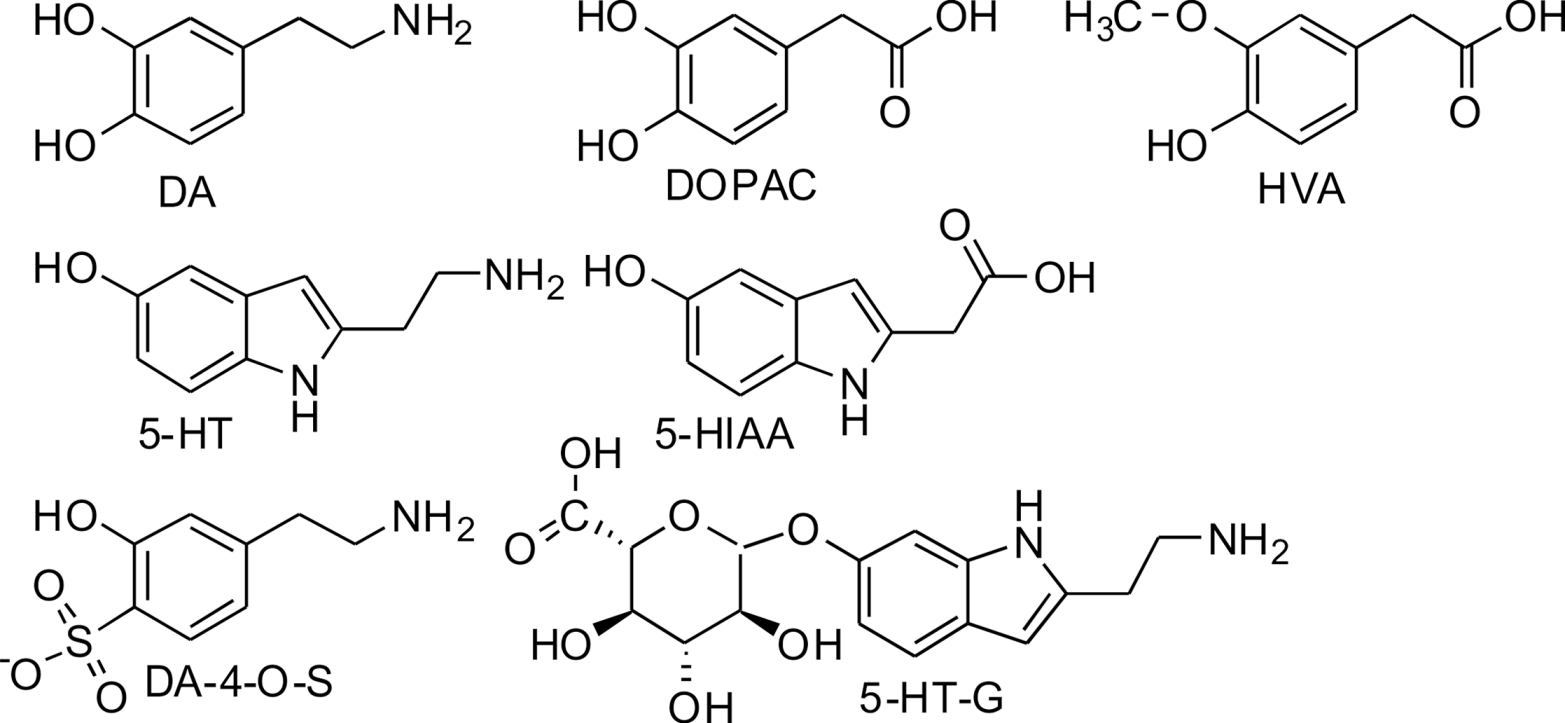
Structures of dopamine and serotonin and their main metabolites.

The purpose of this work was to study the permeation of DA and its sulfonate conjugates, the regioisomers dopamine-3-O-sulfate (DA-3-S) and dopamine-4-O-sulfate (DA-4-S), through the BBB by subcutaneous (s.c.) injections of DA, DA-3-S, and DA-4-S, as well as ^13^C_6_-isotopically labeled DA-3-O-S (^13^DA-3-S) and DA-4-O-S (^13^DA-4-S) in rats. The fate of the injected compounds and their possible effects on the concentrations of dopamine, serotonin, and their metabolites (Fig 1) were analyzed in rat brain microdialysis samples using ultra-performance liquid chromatographic-tandem mass spectrometry (UPLC-MS/MS).

## Materials and Methods

### Reagents and standards

3,4-dihydroxyphenethylamine hydrochloride (DA) and 3-methoxy-4-hydroxy-phenylacetic acid (HVA) were purchased from Sigma-Aldrich (St. Louis, MO). 5-hydroxytryptamine (5-HT), 3,4-dihydroxyphenylacetic acid (DOPAC) and 5-hydroxyindole-3-acetic acid (5-HIAA), formic acid, and dimethyl sulfoxide-d_6_ (CD_3_SOCD_3_) were purchased from Sigma-Aldrich (Steinheim, Germany). ^13^C_6_-labelled dopamine (^13^DA) was purchased from C/D/N isotopes, Quebec, Canada. Acetonitrile (ACN) was purchased from Rathburn (Walkerburn, Scotland). Sulfuric acid (H_2_SO_4_, BDH, 98%) was used in the chemical synthesis of sulfate conjugates. Isoflurane (Vetflurane, Virbac, France) was purchased from the University Pharmacy, Helsinki, Finland. The phase II metabolites of 5-HT, DA, and their phase I metabolites (Table 1), which were used as reference standards in this study, had been synthesized earlier in our laboratory by the methods described in detail elsewhere [23,24,33].

**Table 1:** The analytes, SRM transitions and validation data.

				**Linearity**		**LOD**	**LOQ**	**Repeatability, n = 6**
	**Rt**		**ESI**	**range**		**S/N 3**	**S/N≥10**	**0.5^1^/10^2^/20^3^/ 100^4^**
**Compound**	**min**	**SRM transitions**	**+/-**	**nM**	**r2**	**nM**	**nM**	**nM (rsd %)**
**DA-G**	1.81	330→137 (154, 91)	+	0.5–20	0.995	0.1	0.5	13.0^1^
**DA**	1.98	154→137 (119, 91)	+	0.3–50	0.995	0.2	0.3	11.6^1^
**5-HT-G**	2.69	353→160 (336, 177)	+	0.2–2.5	0.996	0.1	0.2	8.0^1^
**5-HT**	4.17	177→160 (115, 132)	+	0.3–10	0.997	0.2	0.3	7.7^1^
**5-HT-S**	5.23	257→240 (160, 115)	+	1–100	0.999	0.4	1	2.6^2^
**5-HIAA-G**	6.35	366→146 (131, 113)	-	10–750	0.999	5	10	9.6^3^
**HVA-OH-G**	6.74	357→193 (113, 175, 313)	-	1–75	0.999	0.7	1	3.1^3^
**5-HIAA-S**	8.18	270→226 (146, 80)	-	10–500	0.996	5	10	11.6^3^
**5-HIAA**	8.76	190→146 (144, 116)	-	40–750	0.996	10	40	4.2^3^
**DA-4-O-S**	2.68	232→152 (122)	-	0.5–50	0.999	0.25	0.5	5.7^2^
**DA-3-O-S**	2.82	232→152 (122)	-	0.5–125	0.997	0.25	0.5	5.1^2^
**DOPAC-G**	5.47	343→299 (123, 113)	-	20–750	0.999	4	20	7.4^3^
**DOPAC**	5.7	167 → 123 (95)	-	30–1000	0.998	20	30	6.7^4^
**DOPAC-S**	5.99	247→203 (123, 80)	-	10–500	0.998	5	10	nm
**HVA-S**	7.33	261→217 (202, 80)	-	5–500	0.999	2.5	5	nm
**HVA**	9.35	181→137 (122, 105)	-	30–1000	0.999	25	30	7.0^4^
**^13^C-DA-G**	1.81	336→143 (160, 97)	+					
**^13^C-DA**	1.98	160→143 (125, 97)	+					
**^13^C-DA-4-O-S**	2.68	238→158 (128)	-					
**^13^C-DA-3-O-S**	2.82	238→158 (128)	-					
**^13^C-DOPAC-G**	5.47	349→305 (129, 113)	-					
**^13^C-DOPAC**	5.70	173 → 129 (101)	-					
**^13^C-DOPAC-S**	5.99	253→209 (129)	-					
**^13^C-HVA-G**	6.74	363→193 (113, 175, 319)	-					
**^13^C-HVA-S**	7.33	267→223 (208, 80)	-					
**^13^C-HVA**	9.35	187→143 (128)	-					

The concentrations in the repeatability measurements were 0.5, 10, 20, or 100 nM presented as superscripts 1, 2, 3, or 4, respectively.

Ringer’s solution, used for microdialysis and dilution of the standards, contained 147 mM NaCl (Akzo Nobel, Denmark), 1.2 mM CaCl_2_ (J.T.Bakers, Deventer, The Netherlands), 2.7 mM KCl (Sigma-Aldrich, Steinheim, Germany), 1.0 mM MgCl_2_ (Riedel-de-Haehn AG, Seelze, Germany), and 0.04 mM ascorbic acid (University Pharmacy, Helsinki, Finland).

### Chemical synthesis of ^13^C_6_-dopamine sulfates

Cold concentrated H_2_SO_4_ (200 *μ*L) was added to 20 mg of ^13^C_6_-DA HCl. The reaction mixture was kept in ice for 20 minutes and then pipetted over 1 mL of frozen water. The pH of the reaction mixture was adjusted to 3 with 5 M NaOH. The sulfates were fractionated using an Agilent HP 1100 liquid chromatograph (Hewlett-Packard GmbH, Waldbronn, Germany) equipped with a UV diode array detector and fraction collector. A Discovery HS F5 column (150 mm × 4 mm, 3 *μ*m, Sigma-Aldrich, Bellefonte, PA) was used in the purification. Aqueous 0.1% formic acid (A) and ACN containing 0.1% formic acid (B) were used as eluents. A gradient of 5% solvent B from 0–1 min, 5–20% 1–11 min, 20–65% 11–12 min followed by 8 minutes equilibration was used for the fractioning of ^13^DA-3-S and ^13^DA-4-S. The ^13^DA-S regioisomers were not completely baseline separated (R_S_ = 1.0), and were fractionated together. The flow rate was 0.9 mL/min, and a wavelength of 275 nm was used for peak detection. The sulfate fractions, containing both ^13^DA-3-S and ^13^DA-4-S, were evaporated to dryness under vacuum, lyophilized and reconstituted in Ringer’s solution. The synthesis yield was 3.7 mg (15%). As the yield was low, UPLC-MS was used for the confirmation of the structure of ^13^DA-3-S and ^13^DA-4-S. Unlabeled DA-3-S and DA-4-S were synthesized in the same manner, and the synthesis products were characterized by UPLC-MS and NMR.

### Rat brain microdialysis samples

Wistar rats were used at 8–12 weeks of age. The protocols were approved by the National Animal Experiment Board of Finland. The rats were housed in groups of four to five per cage and had free access to chow and water. They were maintained under a 12:12 h light/dark cycle with lights on from 06:00 to 18:00 at an ambient temperature of 20–22 °C before the experiments.

The animals were implanted with a guide cannula (BAS MD-2250, Bioanalytical Systems Inc., IN) using a stereotaxic device (Stoelting, Wood Dale, IL) under isoflurane anesthesia (4.5% during induction for 5 min and then 3.5% during surgery). The guide cannula was aimed above the rat dorsal striatum (A/P +1.0, L/M 2.7, D/V-6.0) according to the atlas by Paxinos and Watson [34]. The cannula was fastened to the skull with dental cement (Aqualox, Voco, Germany). A microdialysis probe (BAS MD-2200, 2 mm membrane, Bioanalytical Systems Inc., IN) was inserted into the striatum through the guide cannula on the morning of the experimental day. Thus, the coordinates of dialysis membranes within the striatal tissue were A/P +1.0, L/M 2.7, D/V-6.0 –-8.0.

The collection of microdialysis samples with 30 min intervals (2.5 *μ*L/min) began 1 h after insertion of the probe. Two baseline samples were collected prior to the injection of a solution of 10 mM DA and 10 mM DA-S (containing the regioisomers DA-3-S and DA-4-S) at a volume of 1 mL/kg body weight (animals 1–3), or 10 mM ^13^DA-S (containing the regiosisomers ^13^DA-3-S and ^13^DA-4-S) at a volume of 1 mL/kg body weight (animals 4–7). After the injections, microdialysis samples were collected for 3 hours. The microdialysis samples were stored in a freezer (-70°C) before analysis with UPLC-MS/MS. The samples were injected as such without sample pretreatment.

### Plasma samples

Immediately after initiation of isoflurane anesthesia, a blood sample was collected from the femoral vein (time-point 0 hours), after which a solution containing 10 mM DA-S (containing the regioisomers DA-3-S and DA-4-S) at a volume of 1 mL/kg body weight was administered by subcutaneous injection. A second blood sample was collected at 0.5 hours, and the final trunk blood sample 1 hour after the DA-S injection. The blood samples were collected in capillary tubes (Microvette CB 300, Sarstedt, Germany) and centrifuged at 4000 g for 10 minutes at 4°C. The supernatant was ultrafiltered (Vivaspin, 10 0000 MWCO PES, Vivascience AG, Hannover, Germany) at 8600 g for 35 min at 4°C prior to analysis by UPLC-MS/MS.

### UPLC-UV and UPLC-MS/MS

The UPLC-MS/MS system used in the analysis consisted of an Aquity UPLC equipped with a photodiode array UV detector (Waters, Milford, MA) coupled to an Agilent 6410 triple quadrupole (Agilent technologies, Santa Clara, CA). The column used was a pentafluorophenyl column (Thermo Scientific Gold PFP Hypersil, 2.1 x 150 mm, 1.9 μm). The eluents used were A: aqueous 0.1% formic acid, and B: acetonitrile and 0.1% formic acid. The gradient used in the analysis of the samples was: B 3% 0–1.5 min, 3–15% 1.6–12 min, 16–65% 13–13.2 min, and 3% 13.5 min, followed by an equilibration of 11.5 min with a total run time of 25 min. The flow rate was 0.3 mL/min, and the column oven temperature was kept at 30°C. The injection volume was 15 μL. The flow from the UPLC was directed to waste for the first 2.1 minutes of the gradient to avoid contamination of the ion source by the salts of the Ringer’s solution.

The purity of the synthesized DA-S was determined by UPLC-UV using Empower 2 software (Build 2154). The Agilent 6410 triple-quadrupole mass spectrometer with an electrospray ion source was used for the verification of the structure of the synthesis products, and for the analysis of the brain microdialysis samples. Nitrogen (Parker Balston N2-22 nitrogen generator, Parker Hannifin Corporation, Haverhill) was used as the nebulizer (40 psi), curtain (12 L/min, 350°C), and collision gas. The fragmentor voltages and collision energies were optimized for each compound, and Agilent MassHunter software versions B.04.00 (quantitative data analysis) and B.03.01 (qualitative data analysis) were used for data acquisition and processing. The quantitative analysis of the brain microdialysis samples was performed in selected reaction monitoring (SRM) mode using positive or negative ion mode (Table 1). The method was validated in terms of linearity, limit of detection (LOD), limit of quantification (LOQ), specificity, and repeatability for each compound.

### NMR spectroscopy

The synthesized and purified compounds were analyzed by nuclear magnetic resonance (NMR) on a Varian Mercury 300 MHz spectrometer (Varian, Palo Alto, CA). ^1^H-spectra were recorded as solutions in DMSO-*d*_6_. Chemical shifts (δ) are given in parts per million (ppm) relative to the NMR solvent signal (DMSO-*d*_6_) 2.50 ppm. As the obtained amounts of ^13^DA-3-S and ^13^DA-4-S were too low for reliable NMR analysis, unlabeled DA-3-S and DA-4-S were synthesized in the same manner and used for NMR characterization.

## Results and Discussion

### ^13^DA-sulfate synthesis

The yield of the chemical synthesis of the mixture of ^13^DA-3-O-S and ^13^DA-4-O-S was 3.7 mg (15%). The purity of the sulfates was estimated by UPLC-UV (diode array detection), and the resulting chromatograms showed over 98% purity. The structures of the synthesized compounds were verified by UPLC-MS and UPLC-MS/MS using negative ion mode. The ion chromatograms showed two peaks, the MS spectra of which showed abundant [M-H]^-^ ions at m/z 238 indicating the correct molecular weights of the synthetized ^13^DA-3-S and ^13^DA-4-S. The product ion spectra of the [M-H]^-^ showed abundant product ions [M-H-SO_3_]^-^ (m/z 158) and minor fragments [M-H-CH_2_NH_2_]^-^ (m/z 128) and [SO_3_]^-^ (m/z 80). Based on UV detection, the mixture of the ^13^DA-S regioisomers contained 53% of ^13^DA-4-S and 47% of ^13^DA-3-S. The spectra of the DA-S regioisomers are in accordance with earlier reported NMR data [33] confirming sulfonation to the hydroxyl groups attached to carbons 3 and 4.

#### Characterization of dopamine sulfates by NMR

The sulfonation sites of the synthesized DA-3- and DA-4-S were verified by NMR. The NMR spectrum was acquired from a mixture of the regioisomers. The transitions are in accordance with earlier reported NMR data of the separated regioisomers [33]. The structures of DA-3- and DA-4-S are presented in Fig 1.

DA-4-O-S: ^1^H NMR (300 MHz, DMSO-d_6_) *δ* 8.00 (br. s, approx.1H), 7.06 (d, *J* = 8.2 Hz, 1H, partially overlapping with DA-4 protons), 6.73 (d, *J* = 1.8 Hz, 1H), 6.64 (d, *J* = 8.1 Hz, 1H), 3.04–2.95 (m, 2H, overlapping with DA-4 protons), 2.76–2.70 (m, 2H, overlapping with DA-4 protons).

DA-3-O-S: ^1^H NMR (300 MHz, DMSO-d_6_) *δ* 8.00 (br. s, approx.1H), 7.05 (m, *J* = 2.2 Hz, 1H, partially overlapping with DA-3 protons), 6.83 (dd, *J* = 8.2, 1.8 Hz, 1H), 6.77 (d, *J* = 8.2 Hz, 1H), 3.04–2.95 (m, 2H, overlapping with DA-3 protons), 2.76–2.70 (m, 2H, overlapping with DA-3 protons).

### UPLC-MS/MS method

The UPLC-MS/MS method was validated in terms of limits of detection (LOD, signal-to-noise ratio (S/N) of 3) and limit of quantification (LOQ, S/N of at least 10), linearity, and repeatability using standards diluted in Ringer’s solution (Table 1). The LODs for DA, 5-HT, and their metabolites varied between 0.1 and 20 nM, indicating good sensitivity of the method and measuring at the same level as presented earlier for these compounds [24,35]. The calibration curves showed good linearity, r^2^ being higher than 0.995 for all the compounds. The repeatability of the method was studied at different concentration levels (0.5, 10, 20, or 100 nM), corresponding to the concentrations of the analytes determined in the brain microdialysis samples (Table 1). The relative standard deviations (% RSD) were below 15%, indicating good repeatability of the analysis. The validation results show that the UPLC-MS/MS method provided direct and reliable analysis of the neurotransmitters, their phase I metabolites and their sulfate and glucuronide conjugates. The ^13^C_6_-isotopically labeled compounds were identified and quantified using the retention times, SRM transitions, and calibration curves of the corresponding unlabeled compounds. The analytes in the brain microdialysates were detected by comparing the retention times and the intensity ratios of at least two SRM pairs for each compound (Table 1) to those of the standards prepared in Ringer’s solution.

**Fig 2:**
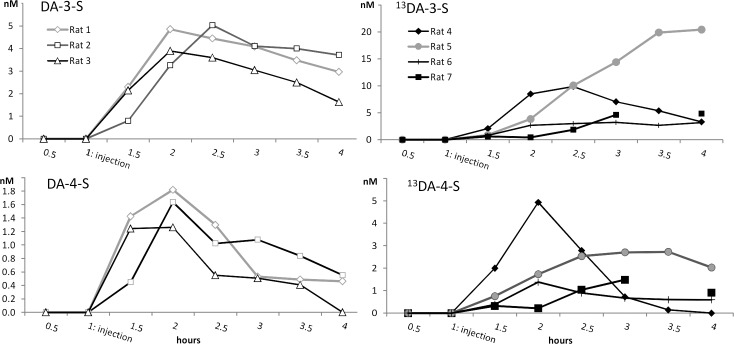
Concentrations of non-labeled and ^13^C_6_-labelled DA-3-S and DA-4-S in rat brain microdialysates after s.c. injections.

Although the UPLC-MS/MS method itself showed good quantitative performance, the biological variation between the rats caused relatively large variations in the concentrations of DA-S in the plasma and microdialysis samples between the rats. The relative standard deviations were about 50–100% between the microdialysis samples (Table 2 and Fig 2) and 20–60% between the plasma samples (Table 3).

**Table 2:** Mean concentrations (nM) and standard deviations between the rats (see Fig 2) for DA-3-S, ^13^DA-3-S, DA-4-S and ^13^DA-4-S at the timepoints of microdialysis sampling.

**DA-3-S**								
Timepoint (h)	**0.5**	**1**	**1.5**	**2**	**2.5**	**3**	**3.5**	**4**
Mean	0.00	0.00	1.75	4.00	4.36	3.75	3.32	2.77
Sd	0.00	0.00	0.82	0.80	0.72	0.61	0.76	1.06
*Rsd %*			*47*	*20*	*17*	*16*	*23*	*38*
**^13^DA-3-S**								
Timepoint (h)	**0.5**	**1**	**1.5**	**2**	**2.5**	**3**	**3.5**	**4**
Mean	0.00	0.00	1.07	3.85	6.18	7.31	9.30	7.93
Sd	0.00	0.00	0.67	3.39	4.37	4.97	9.27	8.36
*Rsd %*			*62*	*88*	*71*	*68*	*100*	*105*
**DA-4-S**								
Timepoint (h)	**0.5**	**1**	**1.5**	**2**	**2.5**	**3**	**3.5**	**4**
Mean	0.00	0.00	1.06	1.58	0.98	0.82	0.58	0.39
Sd	0.00	0.00	0.54	0.27	0.39	0.53	0.23	0.20
*Rsd %*			*50*	*17*	*39*	*64*	*39*	*52*
**^13^DA-4-S**								
Timepoint (h)	**0.5**	**1**	**1.5**	**2**	**2.5**	**3**	**3.5**	**4**
Mean	0.00	0.00	0.86	2.06	1.81	1.39	1.15	0.88
Sd	0.00	0.00	0.78	2.02	0.99	0.95	1.38	0.85
*Rsd %*			*91*	*98*	*54*	*68*	*119*	*97*

**Table 3:** Concentrations of DA-3-S and DA-4-S in plasma samples (nM) before (0 min) and after s.c. injections (30 and 60 min).

	**DA-3-S**			**DA-4-S**		
	0 min	30 min	60 min	0 min	30 min	60 min
**Rat 1**	11.4	14600	10830	0.0	5340	4000
**Rat 2**	nm	17760	7680	nm	10340	2950
**Rat 3**	4.9	13200	9900	2.6	6280	2630
**mean**	8.2	15200	9470	1.3	7320	3180
**sd**	4.6	2320	1620		2660	692
***rsd %***	*57*	*15*	*17*		*36*	*22*

### Permeation of dopamine and dopamine sulfates through the BBB

A mixture containing DA-S (DA-3-S and DA-4-S, 1:1) 2.3 mg/kg and DA 1.5 mg/kg was injected subcutaneously (s.c) and the levels in rat brain were measured by microdialysis samples collected at 30 minutes intervals for 3 hours after administration (n = 3). By also injecting intact DA, the function of the BBB was controlled, as DA does not permeate the BBB. In all three rats the concentrations of DA-3-S and DA-4-S were below the limit of detection before the injection, but clearly increased 30 min after the injection (Fig 2). To confirm the results, the experiment was repeated with a mixture of labelled ^13^DA-S regioisomers (2.4 mg/kg) (n = 4). Again, in all four rats the concentrations of both ^13^DA-3-S and ^13^DA-4-S clearly increased 30 min after the s.c. injection. In contrast to DA-S, the concentrations of intact DA did not change significantly after the injection, measuring at the same levels as reported earlier in rat striatal microdialysis samples (0.7–8 nM) [23,24,36]. These results clearly show that DA-S permeates the intact BBB.

In general, the concentrations of DA-S in rat brain microdialysis samples increased after the s.c. injection, achieved maximum, and then decreased (Fig 2). The concentration profiles of DA-3-S and DA-4-S were somewhat different: the concentrations of DA-3-S were on average about three times higher than those of DA-4-S in all the brain microdialysis samples of the test animals.

DA-S was also quantified in the rat plasma samples collected at the time-points of 0 (injection of DA-S), 30 and 60 min corresponding to the same time points as in the microdialysis experiments. The results showed that the DA-S concentrations in the plasma samples are about 1000 times higher than in the brain microdialysis samples (Tables 2 and 3) indicating that only a small fraction of DA-S is passed through the BBB. Also the possibility that DA-S is transformed during its diffusion to the microdialysis probe cannot be ruled out. As the concentrations of DA-3-S were around 2–3 times higher than the concentrations of DA-4-S also in the plasma samples, this indicates that DA-3-S and DA-4-S permeate the BBB with similar efficiency.

The fate of DA-S in brain was followed by monitoring the possible hydrolysis and metabolite products of dopamine and serotonin (Table 1). The monitoring of ^13^C_6_-labelled DA-S products provides a selective method for the identification of the possible hydrolysis and metabolite products based on the ^13^C_6_-labelled benzene moiety. However, the ^13^C-labelled products presented in Table 1 were not detected in any of the brain microdialysis samples. These results suggest that DA-S permeates the BBB back to the peripheral circulation, or is metabolized by an unexpected mechanism producing metabolites which were not monitored.

The concentration profiles of naturally occurring (non-labelled) dopamine, serotonin, and their metabolism products (Table 1) were studied by microdialysis samples collected before and after the injection of ^13^C_6_-labelled DA-S. The basal concentrations of the naturally occurring analytes (Table 4) measured before the injections are in accordance with earlier published values for rat brain microdialysis samples collected from freely moving rats [23,24,37,38], even though this experiment was performed with anesthetized rats.

**Table 4:** Basal concentrations (nM) of the neurotransmitters and their metabolites in rat brain microdialysis samples before the injection. The concentrations are the average values of the first two time points (0.5 and 1 h) in the experiments.

	Rat 1	Rat 2	Rat 3	Rat 4	Rat 5	Rat 6
5-HT	0.39	0.23*	0.57	1.66	< LOD	0.28
5-HT-G	0.29	0.27	0.17	0.23	0.18	< LOD
5-HIAA	50.4	56.8*	91	101	96.9	102
5-HIAA-S	7.81	10.3	12.9	12.0	11.2	14.4
DA	1.95	0.92	0.79	0.68	0.69	0.63
DA-G	0.24*	0.22*	0.13*	0.17*	< LOD	< LOD
HVA	249	232	394	nm	430	428
HVA-S	35.4	41	85.4	nm	76.5	74.9
DOPAC	387	222	579	nm	511	401
DOPAC-S	166	151	283	nm	243	198

* = concentration below LOQ, estimated by extrapolation of the standard curve. nm = not measured

**Fig 3:**
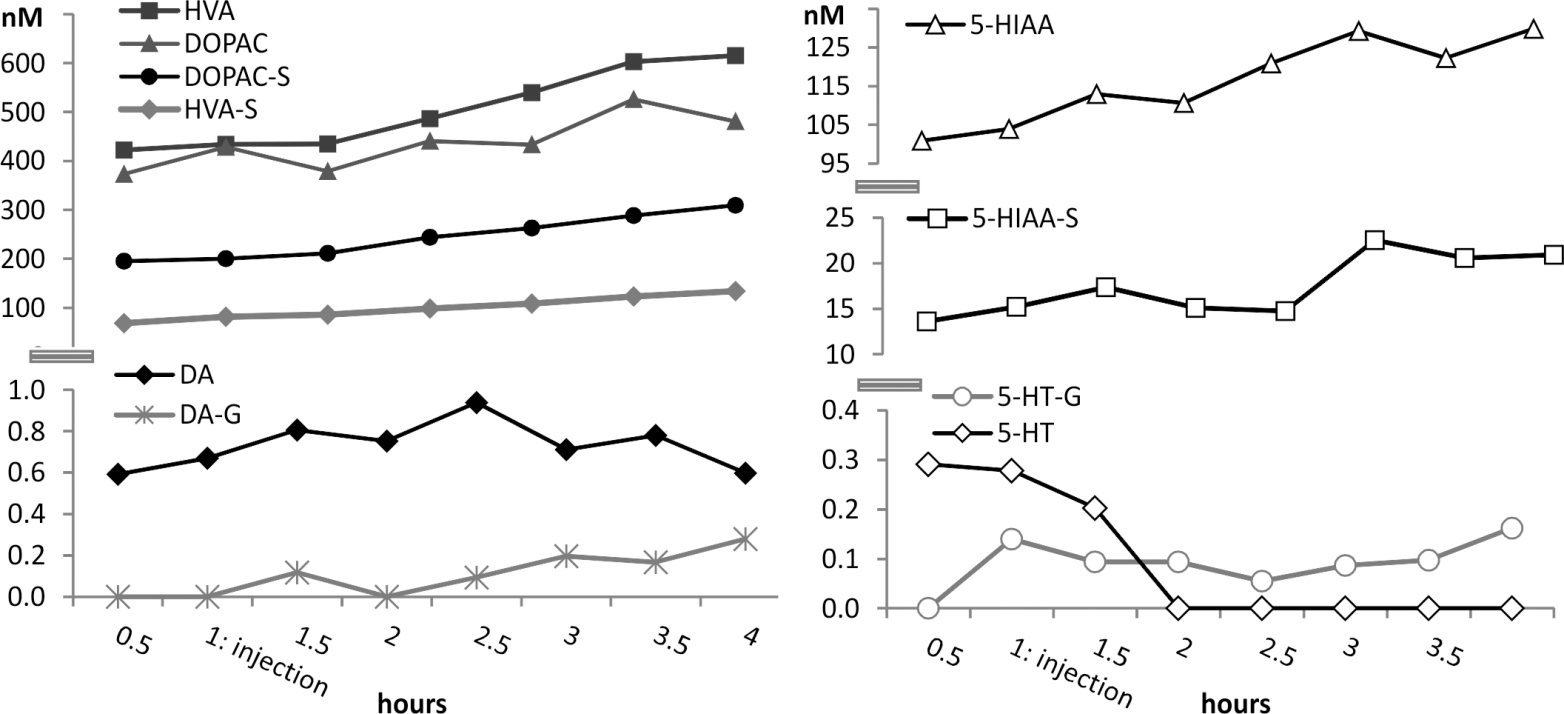
Concentrations of the neurotransmitters (Left: dopamine metabolites, right: serotonin metabolites) during the test in rat brain microdialysates in one of the test animals (Rat 6).

The concentrations of the naturally occurring non-labelled metabolites of dopamine (DA-G, DOPAC, DOPAC-S, HVA and HVA-S) increased after the injections of ^13^C_6_-labelled DA-S. No clear trend in the levels of DA could be observed. Serotonin (5-HT) and its metabolites followed a similar trend: while the 5-HT levels decreased in all animals during the experiment, the concentrations of its metabolites (5-HT-G, 5-HIAA and 5-HIAA-S) increased during the experiment (Fig 3). The injected ^13^DA-S may affect the metabolism process of naturally occurring dopamine and/or other neurotransmitters, but anesthesia itself might also have an impact on the concentration changes. The influence of volatile anesthetics on neurotransmitter levels have been reported earlier: isoflurane has been shown to lower 5-HT levels in rats and mice compared to levels during wakefulness [39,40]. Isoflurane anesthesia has also been shown to increase DA levels at high doses (3% isoflurane), while the levels of DOPAC and HVA increased at all isoflurane concentrations studied [41,42]. Similar effects have been reported for halothane and sevoflurane [43,44]. Based on the literature it seems likely that the fluctuations in neurotransmitter levels are due to anesthesia. Moreover, the isoflurane concentrations had to be slightly adjusted during the experiments, which additionally might have influenced the neurotransmitter levels. However, this does not rule out that the injected DA-S might also have contributed to these level changes.

## Conclusions

The peripheral injections of ^13^C-labelled DA-S followed by analysis of brain microdialysis samples by UPLC-MS/MS provide a reliable method to study the permeation of DA-S through the BBB. The study clearly shows that DA-S, but not DA, is able to permeate the BBB of rats. The result is interesting, since DA-S is more polar than DA and the passive permeation of DA-S through the BBB is not obvious. Therefore, it can be concluded that DA-S most likely permeates the BBB by binding to an active transporter-protein located in the BBB. However, the concentrations of DA-S in the plasma samples after its subcutaneous injection were about 1000 times higher than the concentration in the brain microdialysis samples. Based on the fairly high concentrations in the plasma samples, it is clear that only a small amount of the DA-S in plasma permeates the BBB. However, the results still show that DA-S crosses the BBB but DA itself does not.

Even though the results show that DA-S crosses the BBB, no information on the fate of the permeated DA-S in the brain was acquired. The results showed that DA-S is not hydrolyzed to DA or transformed into any common metabolites of DA with concentrations above the limits of detection of this method. It is therefore possible that DA-S is metabolized by an unexpected mechanism or is pumped out of the brain by active transporters. However, these results obtained from experiments with rats cannot be directly applied to humans. DA-S has earlier been found in human brain microdialysis samples [25] but not in rat brain microdialysis samples, indicating that the concentration of DA-S in human brain is significantly higher than in rat brain. DA-S can be assumed to be biologically less active than DA providing a source for free DA in brain through enzymatic hydrolysis. Therefore, the study of the permeation of DA-S through the human BBB and the fate of DA-S in human brain is of great interest.
